# Photothermally sensitive gold nanocage augments the antitumor efficiency of immune checkpoint blockade in immune “cold” tumors

**DOI:** 10.3389/fimmu.2023.1279221

**Published:** 2023-10-24

**Authors:** Guixiu Xiao, Yujie Zhao, Xueyan Wang, Chuan Zeng, Feng Luo, Jing Jing

**Affiliations:** ^1^ State Key Laboratory of Biotherapy, West China Hospital, Institute for Breast Health Medicine, Sichuan University, Chengdu, Sichuan, China; ^2^ Department of Medical Oncology, Cancer Center, Lung Cancer Center, West China Hospital of Sichuan University, Chengdu, Sichuan, China; ^3^ Radiology Department, Sichuan Jianzhu Hospital, Chengdu, Sichuan, China

**Keywords:** immune “cold” tumor, immune checkpoint blockade, immune microenvironment remodeling, gold nanomaterials, photothermal therapy, melanoma

## Abstract

**Introduction:**

Immune checkpoint blockade (ICB) has revolutionized the therapy landscape of malignancy melanoma. However, the clinical benefits from this regimen remain limited, especially in tumors lacking infiltrated T cells (known as “cold” tumors). Nanoparticle-mediated photothermal therapy (PTT) has demonstrated improved outcomes in the ablation of solid tumors by inducing immunogenic cell death (ICD) and reshaping the tumor immune microenvironment. Therefore, the combination of PTT and ICB is a promising regimen for patients with “cold” tumors.

**Methods:**

A second near-infrared (NIR-II) light-activated gold nanocomposite AuNC@SiO_2_@HA with AuNC as a kernel, silica as shell, and hyaluronic acid (HA) polymer as a targeting molecule, was synthesized for PTT. The fabricated AuNC@SiO_2_@HA nanocomposites underwent various *in vitro* studies to characterize their physicochemical properties, light absorption spectra, photothermal conversion ability, cellular uptake ability, and bioactivities. The synergistic effect of AuNC@SiO_2_@HA-mediated PTT and anti-PD-1 immunotherapy was evaluated using a mouse model of immune “cold” melanoma. The tumor-infiltrating T cells were assessed by immunofluorescence staining and flow cytometry. Furthermore, the mechanism of AuNC@SiO_2_@HA-induced T-cell infiltration was investigated through immunochemistry staining of the ICD-related markers, including HSP70, CRT, and HMGB1. Finally, the safety of AuNC@SiO_2_@HA nanocomposites was evaluated *in vivo*.

**Results:**

The AuNC@SiO_2_@HA nanocomposite with absorption covering 1064 nm was successfully synthesized. The nano-system can be effectively delivered into tumor cells, transform the optical energy into thermal energy upon laser irradiation, and induce tumor cell apoptosis *in vitro*. In an *in vivo* mouse melanoma model, AuNC@SiO_2_@HA nanocomposites significantly induced ICD and T-cell infiltration. The combination of AuNC@SiO_2_@HA and anti-PD-1 antibody synergistically inhibited tumor growth *via* stimulating robust T lymphocyte immune responses.

**Discussion:**

The combination of AuNC@SiO_2_@HA-mediated PTT and anti-PD-1 immunotherapy proposed a neoteric strategy for oncotherapy, which efficiently convert the immune “cold” tumors into “hot” ones.

## 1 Introduction

Immune checkpoint blockade (ICB) therapy has transformed the treatment landscape for malignant tumors, proving to be an effective strategy to treat cancer in many patients (1, 2). This groundbreaking therapy has given hope to countless people and has significantly improved their quality of life. The advancements in immunotherapy are attributed to the knowledge of tumor evasion. Tumors deactivate T/NK cells and evade immune surveillance by engaging immune checkpoint molecules like PD-1:PD-L1, CTLA-4:B7-1/B7-2, HLA-E:NKG2A, CECAM-1:TIM3, etc. (2–6). Blocking these inhibitory immune checkpoints restores the cytotoxicity function of T cells. Over the past few years, the FDA has approved eight monoclonal antibody drugs targeting PD-1:PD-L1 and CTLA-4:B7-1/B7-2, including ipilimumab, pembrolizumab, atezolizumab, nivolumab, durvalumab, etc. (4). Although ICB has shown promising therapeutic potential, only a small population of patients benefits from this treatment regimen, which highlights the requirement of novel treatment strategies to fuel the application of ICB in the clinic (7, 8). The effectiveness of ICB is based on the presence of infiltrated T cells in the tumor microenvironment. Hence, tumors with low infiltrated T cells, also known as “cold” tumors, respond poorly to ICB (9). Generally, immune “cold” tumors have four main characteristics: 1) low tumor immunogenicity, 2) defects in tumor antigen processing and presentation mechanisms, 3) insufficient infiltration of T cells, and 4) an immunosuppressive tumor microenvironment (10–13). Tumors with low immunogenicity cannot be recognized by dendritic cells (DCs), and further activation of T cells cannot be achieved without antigen presentation (10, 14). Thus, the main reason for the failure of ICB therapy is the insufficient infiltration of T cells due to a lack of tumor-specific antigens.

To solve this problem, many strategies have been explored for the purpose of increasing tumor immunogenicity and activating T cells by promoting any of the key steps of antigen release, DC recognition, and antigen presentation (10, 15). Tumor antigen-based immunotherapy has also shown gratifying response rates in various cancers, including vaccines based on tumor-specific antigens, personalized vaccines, whole cancer cell vaccines, and neoantigen-targeting vaccines (16, 17). DC vaccines have also demonstrated the capacity to induce antigen-specific immune responses *in vivo* (15). Chimeric antigen receptor (CAR)-T cell therapies have been developed to stimulate tumor-specific innate and adaptive responses (18). However, huge restrictions still exist when faced with the heterogeneity of patients, such as uncertain dosages and characteristics, as well as expensive costs (19, 20). A more specific and easier method is supposed to weapon the current cancer immunotherapy strategy and improve its efficacy and safety.

In recent years, photothermal therapy (PTT) has emerged as a promising cancer treatment option (21). Photoimmunotherapy based on nanomaterials has specifically shown advantages in promoting the release of tumor-associated antigens (TAA), tumor-specific antigens (TSA), and synergizing immunotherapy (12, 22). Due to the enhanced permeability and retention (EPR) effect, photothermal-sensitive nanoparticles can efficiently accumulate in tumor tissues and convert light energy into heat under near-infrared (NIR) light irradiation (12). This hyperthermia can induce the immunogenic cell death (ICD) of tumor cells, which produce proinflammatory cytokines and damage-associated molecular patterns (DAMPs), resulting in antitumor immune responses (12, 23). Additionally, nanoparticles that mediate PTT can be manipulated to avoid heat damage to normal tissues and have deep tissue penetration ability (24, 25). Gold nanoparticles (AuNPs) are one of the nanomaterials that have been widely applied in PTT due to their great photothermal conversion capabilities and robust plasticity (26). They have superior biocompatibility, biosafety, surface plasmon resonance (SPR), and excellent conversion ability under irradiation (23, 27). More importantly, the strengthened EPR effect and correct size for intravascular transport, make the nanoscaled particles easily accumulate in the tumor microenvironment (28, 29). These outstanding properties of AuNC have led to the approval of the Food and Drug Administration (FDA) for AuNC-based *in vitro* diagnostic systems and clinical trials of AuNC as cancer treatment (30). Recent studies have found that AuNC can produce antitumor immunological effects by generating tumor-associated antigens from ablated tumor cells (31–33).

To improve the effectiveness of anti-PD-1 immunotherapy in immune “cold” tumors, we designed a tumor-targeting nanosystem AuNC@SiO_2_@HA to agitate the immune microenvironment and synergize with ICB in a mouse melanoma characterized by immune “cold” microenvironment. The nanosystem is composed of an AuNC kernel, a silica (SiO_2_) shell, and a target molecule hyaluronic acid (HA). The modification of HA increased the water solubility, EPR effect, and tumor-targeting ability of AuNC. After the second near-infrared region (NIR-II) laser irradiation, AuNC@SiO_2_@HA could effectively absorb and convert light to heat, resulting in significant tumor suppression. Harnessing the potential of PTT, we restored the targeted release of tumor antigens *via* ICD, thereby enhancing the efficacy of ICB therapy in melanomas characterized by a “cold” immune microenvironment. This innovative approach presents an attractive strategy for addressing unresectable deep tumors, particularly those with limited infiltrated T-cell presence, by synergistically combining PTT and ICB therapy.

## 2 Materials and methods

### 2.1 Materials and reagents

Ethylene glycol (EG), sodium chloride (NaCl), silver nitrate (AgNO_3_, purity of 99.8%), polyvinyl pyrrolidone (PVP, with a molecular weight of approximately 55,000), gold chloride trihydrate (HAuCl_4_·3H_2_O, purity of 99%), tetraethoxysilane (TEOS, purity of 98%), and (3-aminopropyl) triethoxysilane (APTES, purity of 98%) were all purchased from Sigma-Aldrich. Nile Red was obtained from Selleck Chemicals LLC, while HA was sourced from J&K Scientific. The anti-PD1 antibody was a generous gift from Conmed Biosciences Inc. Dulbecco’s Modified Eagle’s Medium (DMEM) was procured from Gibco Life Technologies. Streptomycin, penicillin, and trypsin-EDTA were acquired from Merck Millipore. Fetal bovine serum (FBS) was procured from Gibco Life Technologies.

### 2.2 Cells and animals

As previously reported (34, 35), the SMM103 and SMM102 mouse melanoma cells were obtained from transgenic Braf^V600E/wt^, Pten^−/−^, Cdkn2^−/−^ mice by our laboratory. These cell lines were cultured in DMEM supplemented with 10% FBS, 100 U/mL penicillin, and 100 μg/mL streptomycin under a 5% CO_2_ atmosphere at 37°C.

Female C57BL/6 immunocompetent mice aged 6–8 weeks (18–22g) were obtained from Beijing Vital River Laboratory Animal Technology Co., Ltd. (Beijing, China). All experiments on animals were conducted following the approved guidelines of the Ethics Review Committee of Animal Experimentation at Sichuan University. The handling of animals adhered to the standards outlined in the Animal Welfare Act.

### 2.3 Synthesis of gold nanocage (AuNC)

The galvanic replacement method involving HAuCl_4_ and silver nanocube (AgNC) was used to synthesize AuNC (33). To prepare AgNC, 25.8 mL of EG was heated at 150 °C for 1 hour, followed by the addition of an EG solution containing 86 μL of sodium sulfide. The solution was maintained at 150 °C for 9 minutes, and then 6 mL of EG containing 129 mg of PVP was introduced. The AgNC was obtained by rapidly injecting 2.5 mL EG containing 48 mM AgNO_3_ into the reaction system. Subsequently, the AgNCs were purified through centrifugation using deionized water and acetone.

A solution of AgNC (500 μL, 3 nM) in deionized water with 1 mg/mL PVP was heated at 100 °C for 10 minutes. An aqueous solution of 0.5 mM HAuCl_4_ was slowly supplemented to the solution at a suitable rate until the UV spectroscopy appeared with an evident optical absorption peak. The solution was then refluxed for 30 minutes before being allowed to return to room temperature. To eliminate AgCl, PVP, and NaCl, the sample underwent multiple rounds of centrifugation and washing with saturated NaCl solution and water.

### 2.4 Loading of Nile Red on nanoparticles

A 200 µL solution of Nile Red in methanol (5 mg/mL) was slowly added dropwise to a 2 mL solution of AuNC nanoparticles in methanol (5 mg/mL) under dark conditions. The mixture liquid was stirred at 300 rpm for 12 hours. Subsequently, the product AuNC@SiO_2_@NileRed was obtained by centrifugation. The particles were washed three times with saline solution and then subjected to freeze-drying.

### 2.5 Synthesis and characteristics of HA-capped AuNC (AuNC@SiO_2_@HA)

The sol-gel approach was used to fabricate the silica layer (36). Briefly, 5 mL of prepared AuNC was combined with 22.5 mL of isopropyl alcohol. Successively, a solution of 0.06 mL TEOS and water ammonia (0.5 mL, 28 wt%) was incrementally introduced into the blend, which was stirred continuously for half a day at room temperature. This procedure resulted in the formation of AuNC particles enveloped by a silica shell, which were subsequently purified with ethanol through centrifugation at 6000 g for 10 minutes. APTES was used to functionalize AuNC@SiO_2_ with -NH_2_. 100 mg AuNC@SiO_2_ was dispersed in 20 mL of isopropyl alcohol, and then 50 µL of APTES was added. The mixed fluid was stirred and heated at 50°C for 5 h. When the temperature decreased, centrifugation was performed to obtain AuNC@SiO_2_-NH_2_ nanoparticles. Subsequently, HA was coated on AuNC@SiO_2_-NH_2_ nanoparticles by electrostatic adsorption. An aqueous solution of HA (Mw = 1800, concentration of 5 mg/mL) was added dropwise into AuNC@SiO_2_-NH_2_ nanoparticles. The solution was stirred for 120 min, and the AuNC@SiO_2_@HA nanoparticles were acquired through centrifugation at 8000 rpm for 5 minutes.

The characteristics of the nanoparticles were measured through transmission electron microscopy (TEM), UV-vis-NIR absorption spectroscopy, and dynamic light scattering (DLS). The samples were diluted 10 times with water as the dispersion medium and the scattering angles were fixed at 90°. The refractive indices employed in the measurement were set at 1.33300.

### 2.6 The uptake and biodistribution analysis of AuNC@SiO_2_@HA nanocomposites in cells

To detect the uptake capability, the AuNC@SiO_2_@HA particles loaded with the hydrophobic dye Nile Red (AuNC@SiO_2_@HA@NileRed) were incubated with SMM102 and SMM103 cells. First, 5 × 10^4^ cells were added to a 12-well plate. After overnight growth, the cells were incubated with the nanocomposites for one day. Subsequently, the cells were washed with PBS and collected for analysis using flow cytometry.

To observe cellular distribution, cells seeded on slides underwent PBS washing and fixation with 4% formaldehyde for 40 minutes following coincubation with the nanocomposites. Alexa Fluor 488-conjugated wheat germ agglutinin (WGA) and DAPI nuclear dye were used for dying cell membranes and nuclei. Finally, the slides were examined on the confocal microscope, and the obtained images were plotted with Olympus FV1000 confocal software.

### 2.7 Photothermal characterizations and therapeutic efficacy *in vitro*

The AuNC@SiO_2_@HA was dissolved in PBS, resulting in a final concentration of 50 μg/mL within a quartz cuvette. The photothermal conversion capacity was characterized by recording the temperature rise under a 1064 nm illumination (0.5 W/cm^2^) at various time intervals. To validate the photothermal stability, the nanosystem was irradiated and naturally cooled for three cycles to observe the changes in temperature. For the tumor cytotoxicity *in vitro*, cell apoptosis was assessed using the Annexin V-PI/7AAD Apoptosis Detection kit (Beijing 4A Biotech Co., Ltd., #FXP08) 24 hours after treatment with AuNC@SiO_2_@HA along with 1064 nm laser irradiation.

### 2.8 Photothermal characterizations and therapeutic efficacy *in vivo*

For the photothermal assessment *in vivo*, mice bearing subcutaneous SMM102 melanoma were randomly categorized into two groups. When the tumors reached an approximate volume of 150 mm³, the animals received intravascular injections of either saline or the AuNC@SiO_2_@HA nanocomposites. Following a 24-hour injection period, the mice were exposed to a laser (1064 nm, 0.5 W/cm²) at determined time points. The increase in temperature within the tumors was recorded to evaluate the transformation efficiency of AuNC@SiO_2_@HA *in vivo* through an infrared thermal camera.

To study the ability of AuNC@SiO_2_@HA -mediated PTT to transform “cold” tumors into “hot” tumors, mice were subjected to subcutaneous implantation of 1 × 10^5^ melanoma cells. Upon reaching a tumor size of approximately 80 mm³, the mice received specific treatments through tail vein injections every three days (AuNC@SiO_2_@HA) or every six days (anti-PD-1 antibody) (1): saline (2), anti-PD-1 (5 mg/kg) (3), AuNC@SiO_2_@HA with laser (50 mg/kg), and (4) AuNC@SiO_2_@HA + anti-PD-1 + laser. After a 24-hour period, the tumors were subjected to irradiation with a 1064 nm laser at 0.5 W/cm² for 6 minutes. On the 18th day, at the end of the treatment, euthanasia was performed on the mice, and the tumors were harvested for further experiments. The tumor inhibition rate for the experimental group was calculated using the following formula:


$$\begin{array}{c} \text{Tumor Inhibition Rate }(\%)=(\text{Mean Volume of Control Group }–\\ \text{Mean Volume of Experimental Group})/\text{Mean Volume of Control Group x }100\%. \end{array}$$

### 2.9 Safety and toxicity evaluations of AuNC@SiO_2_@HA *in vivo*

To assess the adverse effects and general toxicity of AuNC@SiO_2_@HA, all animals were monitored for observable indicators including appearance, independent activity, and mortality. Following the mice being euthanized, major organs such as the heart, lung, liver, kidney, and spleen were collected, fixed in paraformaldehyde (4%), sectioned, and subjected to hematoxylin and eosin (H&E) staining for histopathological analysis. After the mice treated with different agents were sacrificed, the blood samples and serum were collected for hematological analysis, serological, and biochemical analyses in an automatic analyzer.

### 2.10 Immunohistochemistry and immunofluorescence staining

Tumor tissues were fixed using 10% formalin, embedded in paraffin, and sectioned into pieces measuring 4 μm in thickness. After putting at 60°C for at least 2 h, the tissue sections were dewaxed with xylene and gradient ethanol and then microwaved in EDTA (pH = 9.0) or citric acid (pH = 6.0) for antigen retrieval. The slides were brought to room temperature and washed in PBS. Then, 3% hydrogen peroxide was used for blocking. The slides were incubated overnight with primary antibodies. After that, slides were rinsed and incubated with suitable HRP-conjugated secondary antibodies at room temperature for 30 min. Subsequently, visualization was achieved using DAB (3, 3′-Diaminobenzidine), and the slides were washed, counterstained with hematoxylin, dehydrated using a gradient of ethanol, and finally mounted. For the immunofluorescence staining, slides were incubated with the fluorescently labeled secondary antibody after incubation with the primary antibody and then mounted using DAPI nuclear dye. The acquired pictures were captured using confocal microscopy and analyzed by Image J or Olympus CellSens software.

ImageJ was used to calculate the relative intensity of staining positive cells in IHC and count the number of staining positive cells in the results of IF staining. Then, we calculated the areas of the corresponding picture. The density of CD8^+^ cells was obtained using the following formula:


$$\text{The Density of CD}8^{+}\text{ Cells}=\text{Number of Fluorescent Cells}/\text{Area }({\text{mm}}^{2}).$$

Then we calculated and depicted the average density of cells in each group.

### 2.11 Flow cytometry analysis

Tumor samples were dissected into small pieces using scissors. To digest the tissue fragments into single-cell suspension, DMEM containing type I (1 mg/mL, Gibco) and type IV collagenase (1 mg/mL, Gibco) was employed and cultivated at 37°C for 30 minutes. Then, the single-cell suspension was obtained by filtration with a 70 μm cell filter. After being washed twice with D′-HANKS buffer, the cells were cultivated with Fc-Block to avoid nonspecific binding. The following fluorophore-conjugated antibodies (Biolegend) were used for staining analysis: CD3-Percp-cy5.5 (#100218), CD45-APC-Cy7 (#103154), CD8-PE (#100707), CD44-PE-Cy7 (#103030), CD4-FITC (#100510), CD69-BV710 (#104537), and CD62L-BV605 (#104437). Cells were defined by cell surface molecules as follows (1): CD45^+^CD3^+^presents T cells (2); CD45^+^CD3^+^CD8^+^ indicates CD8^+^ T cells (3); CD45^+^CD3^+^CD4^+^ presents CD4^+^ T cells (4); CD8^+^CD69^+^ indicates activated CD8^+^ cells (5); CD8^+^CD44^+^CD62L^+^ presents memory T cells. After being incubated on ice for 30 minutes and washed, the cells were collected for flow cytometry analysis. The final results were expressed as the proportion of various cell subtypes in CD45^+^ cells.

### 2.12 Statistical analysis

Statistical analysis was conducted with SPSS, version 23 (IBM Corp). For comparisons, the one-way analysis of variance was utilized for multiple groups, while paired t-tests were employed for two groups. Significance levels were established as follows: *P < 0.05; **P < 0.01; ***P < 0.001; ****P < 0.0001, ns, no significance. Graphical representations were created using GraphPad Prism 8.1 (GraphPad Software, Inc., La Jolla, CA, USA).

## 3 Results

### 3.1 Development of photothermal sensitive AuNC@SiO_2_@HA nanoparticles

**Figure 1:**
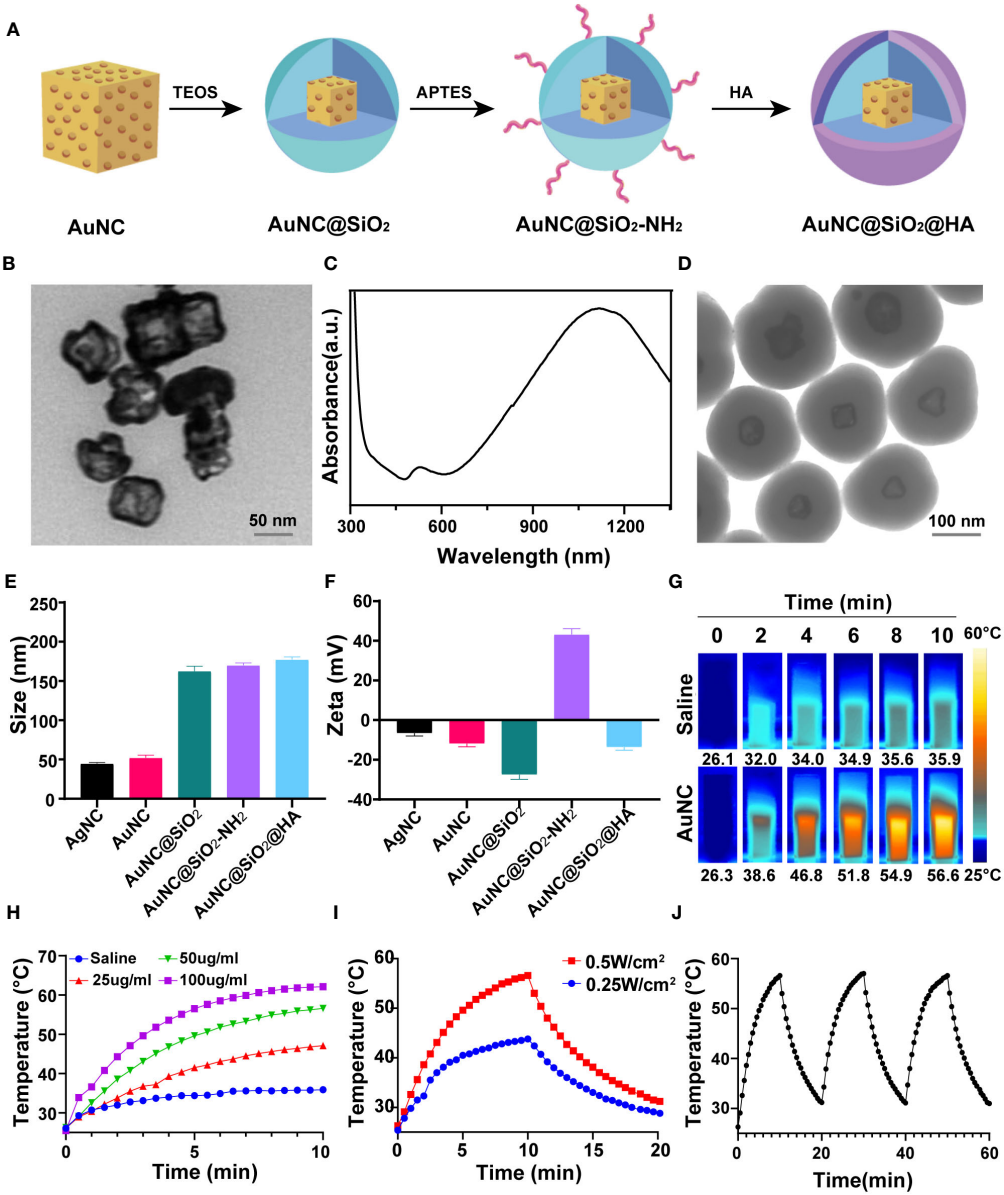
Preparation and characterization of photothermal-responsive nanocomposites AuNC@SiO_2_@HA. **(A)** Diagrammatic representation depicting the fabrication process of AuNC@SiO_2_@HA. **(B)** Transmission electron microscopy (TEM) image of AuNC nanocomposites. Scale bar = 50 nm. **(C)** UV-vis–NIR absorbance spectrum of AuNC@SiO_2_@HA. **(D)** TEM image of AuNC@SiO_2_@HA nanocomposites. Scale bar = 100 nm. **(E)** The particle size (nm) of AgNC, AuNC, AuNC@SiO_2_, AuNC@SiO_2_-NH_2,_ and AuNC@SiO_2_@HA in hydration status. **(F)** The zeta potential of AgNC, AuNC, AuNC@SiO_2_, AuNC@SiO_2_-NH_2_ and AuNC@SiO_2_@HA. **(G, H)** Thermal images **(G)** and corresponding curves **(H)** of AuNC@SiO_2_@HA under irradiation at specified time intervals. **(I)** Photothermal heating and cooling curves of AuNC@SiO_2_@HA (50 μg/mL) under different irradiation conditions. **(J)** Temperature changes of AuNC@SiO_2_@HA over three cycles of irradiation/cooling.

To achieve *in vivo* PTT and modulate the tumor immune microenvironment, we designed and synthesized HA and silica-modified gold nanoparticles (AuNC@SiO_2_@HA) (**Figure 1A**), which were sensitive to NIR light and induced *in vivo* hyperthermia. Firstly, monodispersed AuNC was synthesized through the galvanic replacement reaction as described in Method (37). As shown in the TEM images, the AuNC nanoparticles presented a hollow cubic structure (**Figure 1B**). Then, a condensed silica layer was coated onto the AuNC using the sol-gel method, resulting in AuNC@SiO_2_ nanoparticles. Subsequently, amine groups were introduced on the surface of the nanoparticles by modifying AuNC@SiO_2_ with APTES, yielding AuNC@SiO_2_-NH_2_ nanocomposites. Finally, HA was coated on the surface of the AuNC@SiO_2_-NH_2_ nanocomposites *via* electrostatic adsorption between cations and anions, producing AuNC@SiO_2_-NH_2_@HA nanocomposites (namely AuNC@SiO_2_@HA). The UV-vis-NIR absorption spectra revealed a robust localized surface plasmon resonance (LSPR) peak at 1064 nm for AuNC@SiO_2_@HA, precisely within the NIR-II spectrum (**Figure 1C**). According to the TEM images, the HA-modified silica shell was uniformly coated around the AuNC, and the final size of AuNC@SiO_2_@HA was approximately 180 nm (**Figure 1D**). The findings from DLS showed that both the zeta potential and size of the nanocomposites would dynamically evolve following polymer modification. Compared with the naked AuNC, the particle sizes of AuNC@SiO_2_, AuNC@SiO_2_-NH_2,_ and AuNC@SiO_2_@HA were significantly increased, indicating that silica and HA modification increased the hydrodynamic size of the nanocomposites (**Figure 1E**). Since modification of APTES could introduce positively charged amine groups to AuNC@SiO_2_, the charge would be neutralized by the negatively charged HA polymer coating. The zeta potential changed from negative for AuNC@SiO_2_ to positive for AuNC@SiO_2_-NH_2_, and then returned to negative for AuNC@SiO_2_@HA (**Figure 1F**), indicating that the HA polymer was successfully decorated on the nanoparticles. These results thereby proved that the nanocomposites (AuNC@SiO_2_@HA) were successfully prepared.

To evaluate the photothermal ability of AuNC@SiO_2_@HA, we irradiated the nanocomposites with a 1064 nm laser at indicated time points and measured the temperature changes. The temperature of AuNC@SiO_2_@HA nanocomposite solution (50 µg/mL) increased from 26.3 to 56.6°C after NIR-II irradiation for 10 min (0.5 W/cm^2^, 1064 nm) (**Figure 1G**). In contrast, the negative control sample only increased slightly under the same irradiation conditions. Furthermore, there existed a positive correlation between the temperature rise and the dosage of the nanocomposites (**Figure 1H**). Additionally, we tested the photothermal conversion ability of AuNC@SiO_2_@HA under different doses of laser irradiation. The results showed that both 0.25 W/cm^2^ and 0.5 W/cm^2^ laser irradiation could efficiently elevate the temperature, in which 0.5 W/cm^2^ laser irradiation produced more heat (**Figure 1I**). Upon withdrawal of the laser irradiation, the temperature was dramatically decreased, indicating that the growth in temperature is dependent on the laser irradiation. After proving the excellent photothermal conversion ability of AuNC@SiO_2_@HA, we further evaluated their photothermal stability by repeatedly irradiating the nanocomposites three times. After repeated irradiation, the photothermal conversion ability of AuNC@SiO_2_@HA did not decrease, showing its high photothermal stability (**Figure 1J**). These results showed that AuNC@SiO_2_@HA nanocomposites have the ability to effectively elevate temperature in a time- and dose-dependent manner, displaying promising photothermal performance.

### 3.2 Cellular uptake and photothermal ability of AuNC@SiO_2_@HA

**Figure 2:**
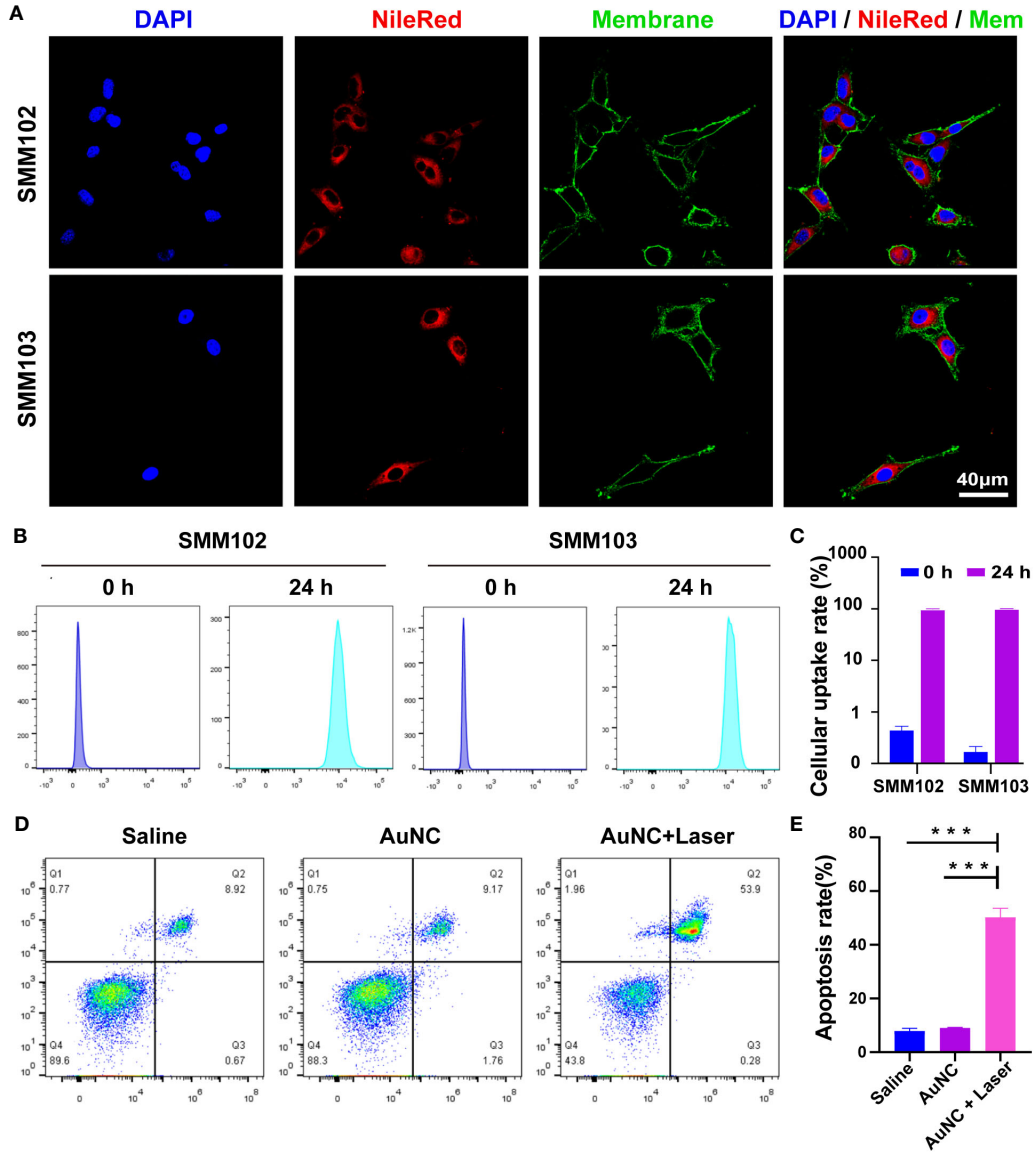
Cellular uptake and bioactivity of AuNC@SiO_2_@HA nanocomposites. **(A)** The fluorescence images display the uptake of Nile Red-labeled AuNC@SiO_2_@HA in SMM102 and SMM103 cells. **(B, C)** SMM102 or SMM103 cells were cultured with AuNC@SiO_2_@HA@NileRed for 24 (h). Then the internalization ability was tested **(B)** and quantified **(C)** by flow cytometry. **(D, E)** The tumor cells were treated with saline or AuNC@SiO_2_@HA with or without laser irradiation (0.5 W/cm^2^). Apoptosis of SMM102 cells was examined by flow cytometric analysis **(D)**, and the apoptosis rate was quantified **(E)**. The results are shown as mean ± SD, ***P < 0.001.

To evaluate the internalization capability of the nanocomposites, a hydrophobic dye (Nile Red) was loaded into the AuNC@SiO_2_@HA nanocomposites (denoted as AuNC@SiO_2_@HA@NileRed). After incubation with tumor cells for one day, the fluorescence signals were obviously filled in the cytoplasm of SMM102 and SMM103 melanoma cells (**Figure 2A**). The red fluorescence signals filled the whole cell, indicating that the particles could be heavily taken up by tumor cells. Flow cytometer was employed to further assess the cellular internalization capabilities. The quantification showed that up to 97.1% of SMM102 and 99.3% of SMM103 cells internalized the particles (**Figures 2B, C**). These results demonstrated the efficient uptake of the AuNC@SiO_2_@HA nanocomposites by the tumor cells.

We then assessed the bioactivities of AuNC@SiO_2_@HA *in vitro*. The SMM102 tumor cells underwent treatment with the AuNC@SiO_2_@HA nanocomposites followed by irradiation with a 1064 nm laser. We observed that AuNC@SiO_2_@HA nanocomposites efficiently induced tumor cell apoptosis after exposure to irradiation (**Figures 2D, E**). The results are consistent with previous studies showing that PTT can initiate cell apoptosis (38). Whereases, there were no differences between the saline group and AuNC@SiO_2_@HA without laser irradiation group, suggesting its low cytotoxicity. These results proved that AuNC@SiO_2_@HA could be ingested efficiently by tumor cells and induce cell apoptosis upon laser irradiation.

**Figure 3:**
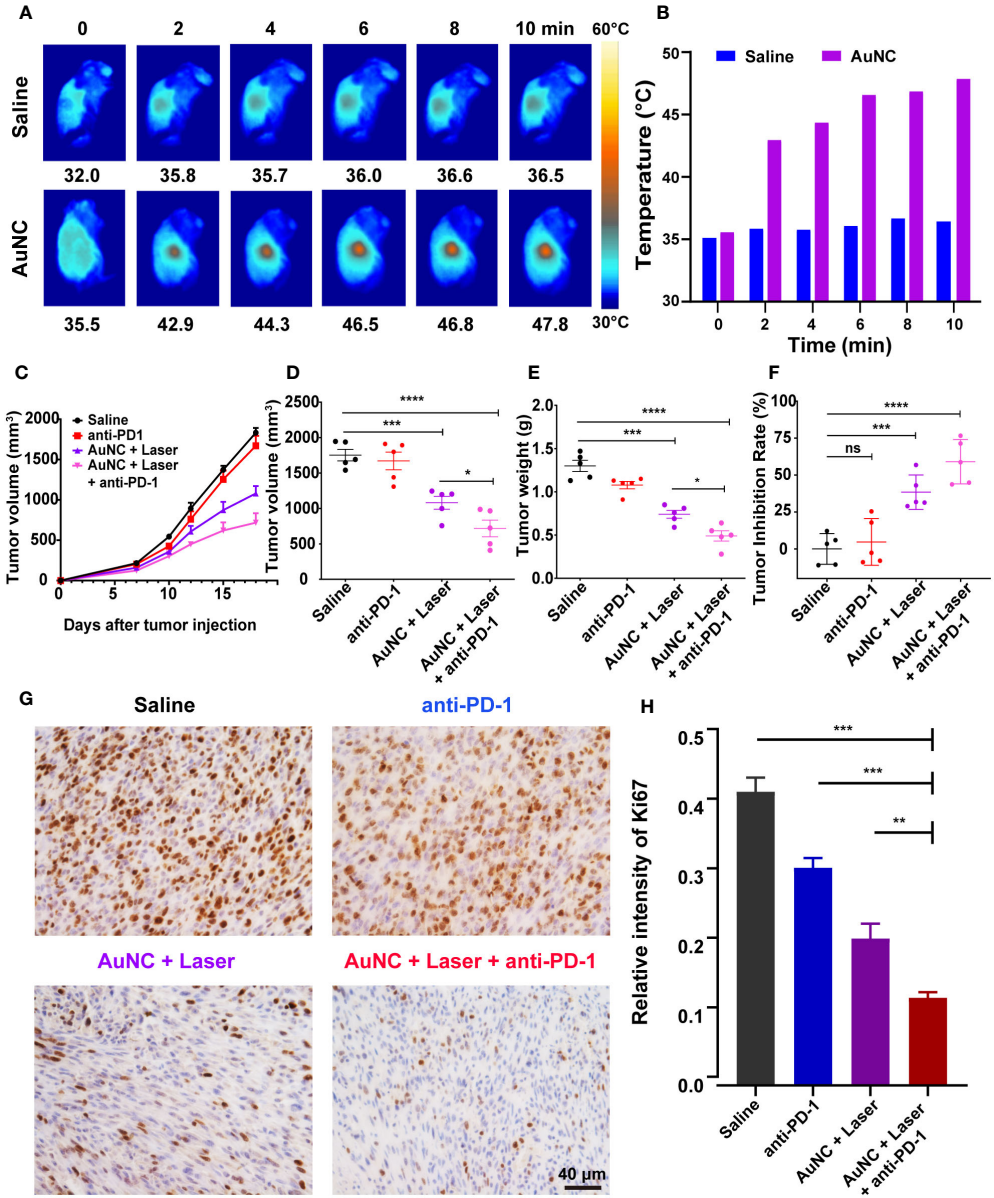
AuNC@SiO_2_@HA nanocomposite-mediated PTT and anti-PD-1 therapy synergistically suppress tumor growth. **(A, B)** IR thermal images **(A)** and temperature **(B)** of melanoma-bearing mice injected with saline or AuNC@SiO_2_@HA nanocomposites under the 1064 nm laser irradiation. **(C–F)** Orthotopic SMM102-bearing mice were administered with saline (control), anti-PD-1, AuNC@SiO_2_@HA with laser, or anti-PD-1 plus AuNC@SiO_2_@HA. The growth curves of tumors were profiled **(C)**. The final volume **(D)** and weight **(E)** of tumors were measured, and the tumor inhibition rate was presented in a scatter diagram **(F)**. **(G, H)** The proliferative status of cancer was determined by Ki-67 IHC. Scale bar = 40 μm **(G)**. The percentage of Ki-67 in each group was quantified **(H)**. The results are shown as mean ± SD (n =5), *P < 0.05, **P < 0.01, ***P < 0.001, ****P < 0.0001, ns, no significance.

The high accessibility and photothermal ability of AuNC@SiO_2_@HA at the tumor site is of great significance for effective PTT. The *in vivo* photothermal ability was assessed in tumor-bearing mice injected with the AuNC@SiO_2_@HA nanocomposites (50 mg/kg). After a 24-hour interval postinjection, the tumor sites underwent illumination for durations of 0, 2, 4, 6, 8, and 10 minutes. The results illustrated a progressive temperature increase in tumors treated with the nanocomposites, elevating from 35.5°C to 47.8°C, significantly surpassing the temperatures observed in the saline group at each time point (**Figures 3A, B**). After being illustrated for 2-6 min, the tumors treated with nanocomposites have reached the ideal temperature for hyperthermia therapy, which is enough to trigger cell apoptosis and can also be well-tolerated (39, 40). In order to optimize treatment duration, enhance therapeutic efficacy, and mitigate the risk of skin overheating, each mouse in the phototherapy group received laser irradiation with a dosage of 0.5 W/cm² for 6 minutes in the subsequent animal experiments. The results indicate that the AuNC@SiO_2_@HA nanomaterials efficiently home in cancer and motivate robust thermal energy after irradiation.

### 3.3 AuNC@SiO_2_@HA -improved the curative effect of anti-PD-1 in immune “cold” tumors

Based on the performance of photothermal effect and tumor-specific cell uptake, we proceeded to assess the *in vivo* antitumor efficacy of AuNC@SiO_2_@HA. SMM102 is a melanoma cell line characterized by low lymphocyte infiltration (“cold” tumor environment) that has been verified in previous articles (33, 41). To further determine the treatment effect of AuNC@SiO_2_@HA, SMM102 was used to establish a mouse model resistant to ICB. After establishing subcutaneous xenografts of SMM102 cells in immune-competent mice, the subjects received different treatments: saline, anti-PD-1, AuNC@SiO_2_@HA with laser exposure, and a combination of anti-PD-1 with AuNC@SiO_2_@HA and laser exposure. The progression of tumor growth was tracked and graphed during the entire period (**Figure 3C**). Upon completion of the treatment on day 18, tumor volumes and weights were calculated after dissection (**Figures 3D, E**). The results showed that anti-PD-1 alone had very limited effects on tumors, while the tumor responded very well to the therapy of AuNC@SiO_2_@HA with laser irradiation. The more exciting result is that obvious inhibition of the tumor was evident within the group treated with anti-PD-1 plus AuNC@SiO_2_@HA with laser irradiation, indicating that the combination therapy could play a more powerful antitumor effect. The tumor weight was consistent with the results of the growth curves. The tumor inhibition rates of PTT alone and PTT plus ICB were 38.4% and 59.0%, respectively (**Figure 3F**). Furthermore, the proliferation status of the tumor was assessed through IHC staining of Ki-67 (**Figure 3G**). Similar to the saline group, tumors treated with anti-PD-1 showed a high proportion of Ki-67^+^ cells. In addition, both the percentage and density of Ki-67^+^ cells were consistent with the anterior results, again confirming the tumor suppression of the combined therapy (**Figure 3H**).

### 3.4 AuNC@SiO_2_@HA enhanced anti-PD-1 immunotherapy by increasing the number of T cells inside tumors

**Figure 4:**
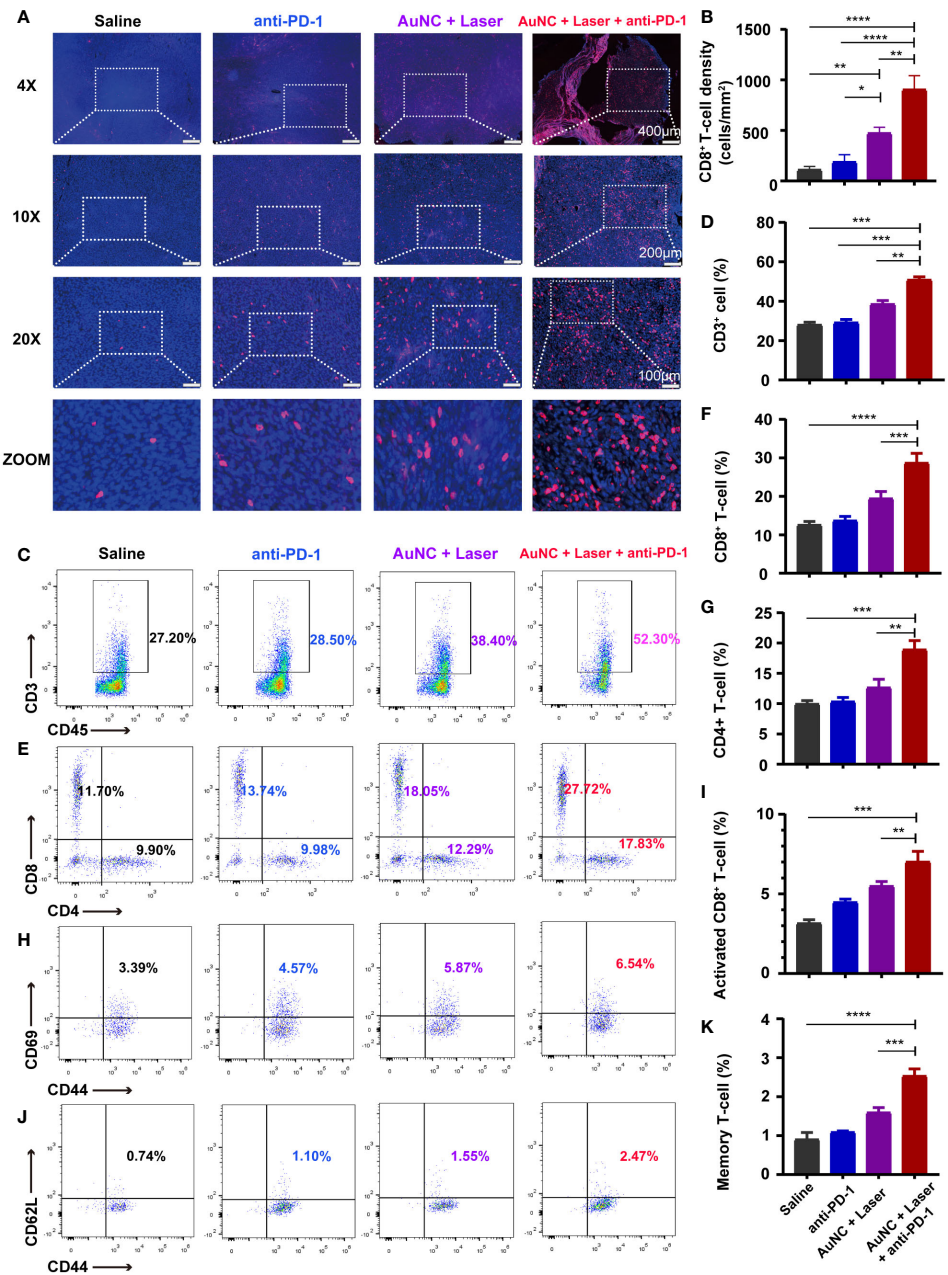
AuNC@SiO_2_@HA nanocomposites synergize with immune checkpoint blockade (ICB) by increasing the T cells inside tumors. **(A)** Images of permeable CD8^+^ T cells in the microenvironment. Scale bars are 400 μm, 200 μm, and 100 μm in the 4-fold, 10-fold, and 20-fold enlarged pictures, respectively. **(B)** Quantification of the densities of CD8^+^ T cells. **(C–K)** The proportion of tumor-infiltrating CD45^+^CD3^+^ immunocytes **(C, D)**, cytotoxic lymphocytes (CTLs) [CD3^+^CD8^+^, **(E, F)**], CD4^+^ T cells [CD3^+^CD4^+^, **(E, G)**], activated CTLs [CD8^+^CD69^+^CD44^+^, **(H, I)**] and memory T cells [CD8^+^CD62L^+^CD44^+^, **(J, K)**] in all groups. The statistical results are presented as mean ± SD, *P < 0.05, **P < 0.01, and ***P < 0.001, ****P < 0.0001.

To delve into the mechanism behind the synergistic treatment, we first detected the changes in T cells within the tumor microenvironment. All tumors were dissected and the tumor-infiltrating lymphocytes (TILs) inside tumor tissue were detected and quantified by immunofluorescence staining (**Figure 4A**). Compared to the control group, the anti-PD-1 group did not show a marked increase in the density of CD8^+^ T cells. The hyperthermia induced by AuNC@SiO_2_@HA effectively facilitated the permeation of CD8^+^ T cells, an enhancement that was notably potentiated by PD-1 blockade (anti-PD-1 combined with AuNC@SiO_2_@HA and laser irradiation) (**Figure 4B**). Excitedly, the CD8^+^ T cells not only proliferated in number but also infiltrated deeply into the tumor mass instead of being limited to the surface. The deep location of the T cells represented its high-efficiency status, which was more conducive to exerting antitumor effects (42). There was a question about whether nanomaterials are engulfed to affect the function of other immunocytes. In our results, the increase in the number and infiltration depth of TILs indicated the safety of the nanocomposites, which caused no damage to the function of CD8^+^ T cells. Taken together, tumor suppression after the combinatorial regimen was mediated by TILs.

To further substantiate the role of immunocytes in this combined strategy, the flow cytometric analysis was used to characterize and quantify the subpopulations of tumor-infiltrating T cells in various groups. The presence of CD3^+^ lymphocytes was notably greater in tumors treated with the combined regimen than in those treated with anti-PD-1 or AuNC@SiO_2_@HA with laser irradiation alone (**Figures 4C, D**). In alignment with the findings from immunofluorescence, the cytotoxic lymphocytes (CTLs, CD3^+^CD8^+^) were significantly promoted by the combinatorial regimen (**Figures 4E, F**). CD4^+^ T cells also showed an increasing trend (**Figures 4E, G**). Among them, the activated CTLs (CD8^+^CD44^+^CD69^+^) exhibited consistent and obvious increases, confirming their leading role in antitumor immune response (**Figures 4H, I**). A plausible explanation is that PTT triggers the ICD of tumor cells, which promotes the release of tumor-specific antigens and T cell infiltration. Moreover, the increase in memory CD8^+^ T lymphocytes (CD8^+^CD44^+^CD62L^−^) was slight compared with others (**Figures 4J, K**). The high abundance of CTLs with killing function in tumor tissues is a good prognostic indicator, which has also been confirmed in previous animal experiments. The results suggested that the combined approach of PTT and ICB could turn “cold” tumors into “hot” ones, providing a feasible method for tumors resistant to ICB.

### 3.5 AuNC@SiO_2_@HA-mediated PTT promoted TILs *via* inducing the ICD of tumor cells

**Figure 5:**
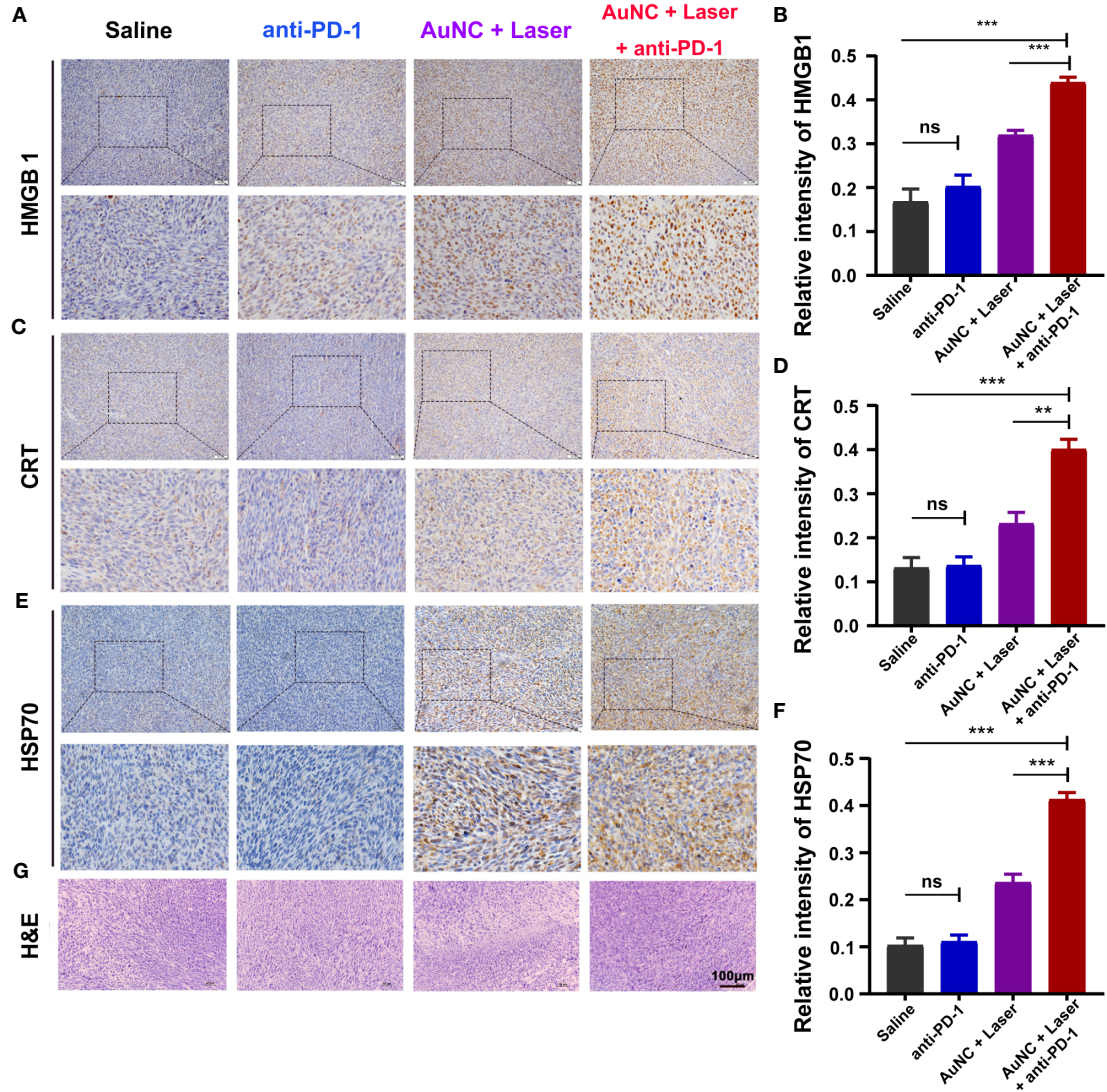
AuNC@SiO_2_@HA nanocomposite-mediated PTT promotes TILs by inducing the immunogenic cell death (ICD) of tumor cells. **(A, B)** High mobility group protein B1 (HMGB1) was analyzed by IHC staining **(A)** and the relative intensity was quantified **(B)**. **(C, D)** The expression of calreticulin (CRT) was evaluated by IHC **(C)** and the relative expression was quantified **(D)**. **(E, F)** Heat shock protein 70 (HSP70) was analyzed **(E)** and quantified **(F)** by the same experiment. **(G)** H&E staining of the tumor tissues. Scale bar = 100 μm. The quantitative results are shown as mean ± SD, **P < 0.01, and ***P < 0.001, ns, no significance.

Injured cells would show molecules on their surface, commonly referred to as DAMPs, which work as danger signals or adjuvants for the innate immune system (43, 44). These DAMPs can be either secreted, as in the case of high mobility group protein B1 (HMGB1), or presented on the plasma membrane, as is seen with calreticulin (CRT) and heat shock protein 70 (HSP70) (43). In order to verify our hypothesis that the infiltrating lymphocytes are attracted by PTT-mediated ICD, HMGB1, CRT, and HSP70 in tumors were evaluated by IHC. The expression of HMGB1 was higher in tumors treated with AuNC@SiO_2_@HA and laser irradiation compared with those without nanocomposites (**Figures 5A, B**). The CRT-positive cells also accounted for a larger proportion in the group with PPT (**Figures 5C, D**). Similarly, HSP70 appeared significantly in tumors treated with AuNC@SiO_2_@HA with laser irradiation (**Figures 5E, F**). There was no apparent difference in these DAMPs between the saline group and anti-PD-1, indicating that SMM102 did not respond to ICB therapy. All the molecular indicators presented stronger expression in the combined regimen group than in the group that received PTT alone, showing a remarkable synergistic effect of PTT and ICB. As we expected, the combinatorial regimen dramatically increased the expression of HMGB1, CRT, and HSP70, indicating that ICD was further enhanced by anti-PD-1 antibodies.

At the same time, we examined the degree of tissue necrosis in each group through H&E staining (**Figure 5G**). The results displayed that there were tightly and regularly packed spherical tumor cells in the saline and anti-PD-1 groups, while the combinational regimen group showed significantly decreased cellularity. Typical apoptotic and necrotic characteristics, including tissue fibrosis, nucleus shrinkage, dissolution, and fragmentation were observed in the combinational regimen group. Thus, the administration of ICB and PTT treatment induced the ICD of tumors and facilitated the release of neoantigens, synergistically enhancing the overall benefits of ICB together.

### 3.6 The safety evolution of AuNC@SiO_2_@HA

**Figure 6:**
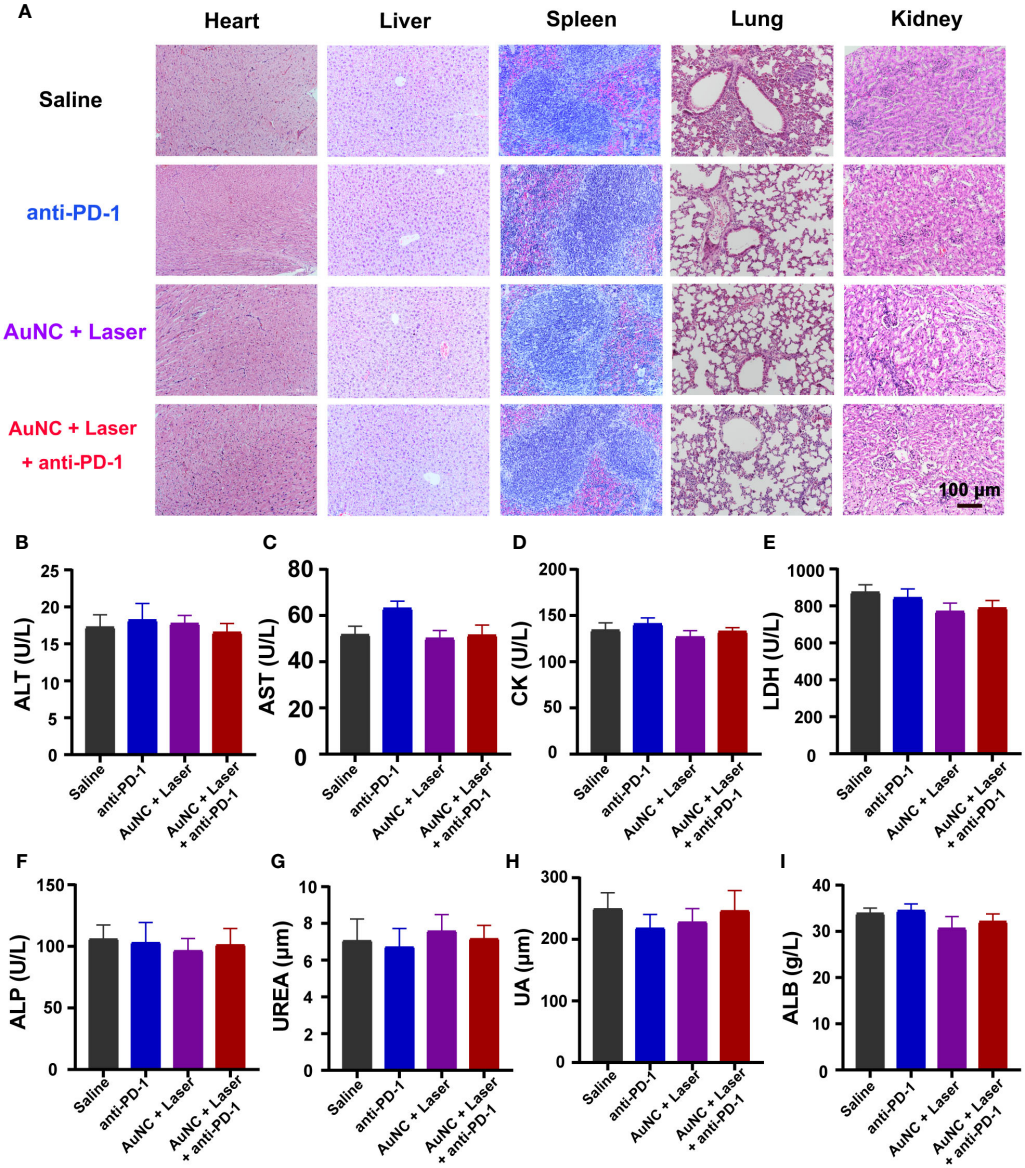
Safety evaluation of AuNC@SiO_2_@HA nanocomposites. **(A)** H&E staining images of different organs of mice in each group. Scale bar = 100 μm. **(B–I)** Hematological analysis evaluated systemic toxicity. Data are presented as mean ± SD (n = 6-8). ALT, aspartate transaminase; AST, aspartate aminotransferase; CK, creatine kinase; LDH, lactate dehydrogenase; ALP, alkaline phosphatase; UREA, urea; UA, uric acid; ALB, albumin.

From the perspective of safety, systemic toxicity was examined in mice. Throughout the duration of the period, no noticeable change in appearance, no significant adverse reaction, and no death were found across all groups. Notably, histopathological examination by H&E staining revealed the absence of discernible pathological presentations in the heart, spleen, liver, kidney, and lung of mice administrated with or without anti-PD-1 and AuNC@SiO_2_@HA with laser irradiation (**Figure 6A**). To further identify the potential side effects, blood samples were collected from the mice for the purpose of performing a hematological analysis and serum biochemical profile testing. For the biomarkers, alanine aminotransaminase (ALT), alkaline phosphatase (ALP), aspartate transaminase (AST), albumin (ALB), and lactate dehydrogenase (LDH) served as an index of liver status; creatine kinase (CK) was employed for diagnosing cardiac conditions; and the change in uric acid (UA) and blood urea was indicative of kidney injury. As shown in **Figures 6B–I**, all the biochemical indices within the drug-treated groups were at normal levels, with no discernible differences from the control group. The data indicated that the treatment had no effects on hepatic function, renal function, or the blood system, further demonstrating the biosecurity of our therapeutic approach.

## 4 Discussion

Therapeutic antibodies that block the PD-1/PD-L1 pathway can induce continuous and powerful reactions in patients with various cancers, however, only a subset of patients benefit from this treatment regimen. The tremendous hurdle for ICB lies in insufficient tumor antigen presentation which leads to limited T cells in the tumor microenvironment (namely, an immune “cold” tumor) (41, 45, 46). In order to improve the efficacy of ICB and reprogram a “cold” immune microenvironment into a “hot” one, finding a simple and safe method to increase the number of infiltrating T cells and promote the release of tumor antigens is important. Thus, we designed an HA-modified AuNC nanocomposite with ingenious electrostatic adsorption, which had greater cancer-targeting ability *via* both active and passive pathways (47). What is more exciting is that the combination treatment exhibited outstanding synergistic tumor inhibition effects both *in vitro* and in mouse models of low immunocyte infiltration. Besides, we demonstrated that upon NIR-II laser irradiation, the nanocomposites induced strong ICD and recruited massive T-effector cells to the deep intratumor. After co-administration, the therapeutic effects of both anti-PD-1 and nanocomposites were magnified, causing more pronounced tumor regression. In summary, PTT triggered ICD and prompted T-cell infiltration, thus synergistically augmenting the therapeutic effect of the PD-1 antibody in “cold” tumors.

Materials with high conversion efficiency have the capacity to generate thermal energy when exposed to external light sources, which is the basis of PTT to kill tumor cells (48–51). Once exposed to irradiation with a specific wavelength, the PTT agents absorb photon energy and migrate from the ground singlet state (S_0_) to an excited singlet state S_1_ (12). When the agents return to the S_0_ state from the S_1_ state, they must undergo vibrational relaxation that results in collisions between the excited photothermal agents and their surrounding molecules. This increased kinetic energy improves the surrounding microenvironment temperature, which will accelerate the permeability of drugs, cause tumor vessel necrosis, and even trigger the instant cell death of tumors. When thermal effects induce the death of a tumor, the cells will express or release tumor-associated antigens and DAMPs, which serve as an “eat me” signal for recruiting and activating antigen-presenting cells (APCs) and further initiate a T cell-mediated immune response to eradicate cancer cells (12, 52).

Emerging research has shown that NIR-II has the advantages of less energy dissipation, higher spatial resolution, and stronger tissue penetration in comparison to traditional NIR-I regions (53–55). Due to the self-thermalization effect stemming from electron-photon scattering and the tunable localized surface plasmon resonance (LSPR) arising from the resonant collective oscillation of conduction electrons, Au holds significant promise as a NIR-II PTT agent (56). Jia and coworkers used AMBI, an organic thiol molecule, to change the emerging Au domains on the Au nanorods, making their LSPRs continuously and effectively tuned within the visible-NIR spectral range (57). In another study, robust intraparticle plasmonic coupling among branches led to an intense and even absorption, endowing Au plasmonic blackbodies (AuPB) with a notable photothermal efficiency exceeding 80% when exposed to 1064 nm photoirradiation (58). Similarly, we fabricated the AuNC through the galvanic replacement reaction between AgNC and HAuCl_4_, resulting in a surface plasmon resonance (SPR) peak within the NIR-II window and displaying remarkable photothermal conversion efficiency. PTT based on NIR-II provides a safer and more effective strategy for patients with unresectable deep cancer.

Gold-based nanomaterials have found extensive application in PTT owing to their good stability, excellent photothermal conversion, near-infrared LSPR peak tunability, and accessible surface functionalization under irradiation (23, 27). However, AuNC lacking surface modification exhibits instability under physiological conditions, rendering them unsuitable for *in vivo* drug delivery (59). To improve the water solubility, biocompatibility, circulation time, and immune escape ability, AuNC have been coated with cell membranes, liposomes, etc. (60, 61). Among all the materials, silica nanoparticles exhibit favorable biocompatibility, degradability, and great chemical and biological robustness (34, 62, 63). In this way, a silica coating was employed to enhance biocompatibility and bioavailability, which imparts great plasticity to the surface modification. The modification of targeted polymers on the surface of silica has been used to improve the enrichment ability of nanoparticles in tumors (64, 65). As an authorized ligand for cell surface receptors such as CD168 and CD44, HA is often used for surface modification of materials to target tumors or inflammatory tissue (66, 67). HA is a nonsulfated and negatively charged glycosaminoglycan with the advantages of nonimmunogenicity, hydrophilicity, biocompatibility, and biodegradability (66). After APTES modification, the positively charged amino groups were introduced on the surface of silica, which can interact with HA *via* electrostatic adsorption. Consequently, the HA-modified AuNC@SiO_2_ nanoparticles are endowed with tumor-targeting ability (68). The temperature at the tumor site rose rapidly under NIR-II laser exposure, confirming the accumulation of AuNC@SiO_2_@HA. In line with this, noticeable suppression of tumor growth was evident in the group receiving combination therapy, indicating the enrichment and antitumor effects of the nanocomposites.

Compared with conventional thermal therapy, the AuNC@SiO_2_@HA nanocomposites exhibited superiority in both biosafety and tumor suppression effects. The systemic immune response triggered by the photothermal effect could be applied in multiple or metastatic tumors. Besides, the advantages of less tissue scattering or absorption, decreased interference, and deeper penetration into biological tissues by fluorescent proteins make NIR-II light a great superior for use in PPT of deep tumors (69). What’s more, the solid silica layer can be loaded with various drugs by etching technology (22, 33, 34, 62). The current silica shell permits the production of materials with a variety of sizes and structures, allowing fine-tuning of the final nanosystem for better drug loading and tumor targeting. In summary, the plasticity, safety, and stability of AuNC nanomaterials determine their clinical accessibility and are expected to achieve clinical transformation in both diagnostic imaging and cancer therapies.

Based on the above advantages, nanoparticle-mediated PTT has become widely used in clinical practice (70–72). In a study, patients with low- to medium-risk localized prostate cancer were treated with gold-silica nanoshell-based magnetic resonance-ultrasound fusion imaging-guided PTT, and 86.7% of them did not show detectable signs within a year (73). Moreover, interventional PTT has achieved a 25% higher median survival rate with complete ablation by one-time intervention when compared to clinical iodine-125 (^125^I) interstitial brachytherapy (74). Furthermore, many studies have proven that PTT can synergize with ICB treatment by inducing the tumor-associated antigen release (12, 75, 76). Taken together, the combination of the two therapies provides a novel cancer treatment strategy with great potential for further clinical applications.

## 5 Conclusion

During the past years, although ICB immunotherapy has greatly enhanced the survival rates of cancer patients, some of the population still cannot benefit from this magical regimen due to the limited T cells inside tumor tissue. To enhance the efficiency of immunotherapy and change the tumor immunosuppressive microenvironment, we developed the NIR-II light-activated gold nanocomposite AuNC@SiO_2_@HA with AuNC as a kernel, silica as a shell, and HA polymer as a targeting molecule. The *in vitro* studies proved that the nanosystem can be effectively taken up by tumor cells, transform the optical energy into heat energy under irradiation, and result in tumor cell apoptosis. The outcomes of *in vivo* investigations revealed that the laser-irradiated nanoparticles can effectively impede tumor growth by inducing hyperthermia. The alliance of anti-PD-1 antibodies with AuNC@SiO_2_@HA nanocomposites synergistically restrains the development of immune “cold” tumors. Mechanistic studies demonstrated that this combo regimen can efficiently induce ICD and lymphocyte infiltration, transforming “cold” tumors into “hot” ones. Taken together, our exploration not only developed a novel NIR-II laser-sensitive photothermal nanocomposites AuNC@SiO_2_@HA but also proposed a novel method for the therapy of immune “cold” tumors in clinics.

## Data availability statement

The raw data supporting the conclusions of this article will be made available by the authors, without undue reservation.

## Ethics statement

The animal study was approved by Ethics Review Committee of Animal Experimentation of Sichuan University. The study was conducted in accordance with the local legislation and institutional requirements.

## Author contributions

GX: Conceptualization, Data curation, Formal Analysis, Investigation, Methodology, Software, Writing – original draft. YZ: Data curation, Formal Analysis, Investigation, Methodology, Software, Visualization, Writing – original draft. XW: Conceptualization, Writing – original draft, Investigation, Software. CZ: Writing – original draft, Formal Analysis, Visualization. FL: Supervision, Writing – review & editing. JJ: Supervision, Writing – review & editing, Project administration, Validation.
